# Autoantibodies against a 43 KDa Muscle Protein in Inclusion Body Myositis

**DOI:** 10.1371/journal.pone.0020266

**Published:** 2011-05-23

**Authors:** Mohammad Salajegheh, Theresa Lam, Steven A. Greenberg

**Affiliations:** 1 Department of Neurology, Brigham and Women's Hospital, Boston, Massachusetts, United States of America; 2 Children's Hospital Informatics Program, Children's Hospital Boston, Boston, Massachusetts, United States of America; 3 Harvard Medical School, Boston, Massachusetts, United States of America; National Institute of Dental and Craniofacial Research, United States of America

## Abstract

**Background:**

Inclusion body myositis (IBM) is a poorly understood and refractory autoimmune muscle disease. Though widely believed to have no significant humoral autoimmunity, we sought to identify novel autoantibodies with high specificity for this disease.

**Methodology/Principal Findings:**

Plasma autoantibodies from 65 people, including 25 with IBM, were analyzed by immunoblots against normal human muscle. Thirteen of 25 (52%) IBM patient samples recognized an approximately 43 kDa muscle protein. No other disease (N = 25) or healthy volunteer (N = 15) samples recognized this protein.

**Conclusions:**

Circulating antibodies against a 43-kDa muscle autoantigen may lead to the discovery of a novel biomarker for IBM. Its high specificity for IBM among patients with autoimmune myopathies furthermore suggests a relationship to disease pathogenesis.

## Introduction

The inflammatory myopathies are autoimmune diseases of skeletal muscle, and consist of three major subtypes: dermatomyositis, polymyositis, and inclusion body myositis (IBM). Circulating autoantibodies have been detected in dermatomyositis and polymyositis (reviewed in [1]) and sought in IBM [2]; however, none have been reported as prominently present in, or specific to, IBM [3].

Since 1984 [4], IBM has been believed to be a cytotoxic T-cell mediated disease with no humoral autoimmunity. Microarray studies reported in 2001, surprisingly showed that the most abundantly present transcripts in IBM muscle samples compared to normal muscle were immunoglobulin transcripts, unique to the B cell lineage [5], [6]. This finding led to the demonstration in IBM muscle of abundant plasma cells [7] with immunoglobulin gene rearrangements, characteristic of clonal expansion in response to local antigen stimulation [8], as well as the presence of a permissive environment for ectopic lymphoid structures suggestive of local maturation of B cells in muscle [9]. The presence of this recently elucidated B cell immunopathogenesis provided rationale for searching for circulating autoantibodies.

In this study we report identification of a circulating autoantibody against a 43-kDa muscle autoantigen that is specific to IBM among other patients with autoimmune myopathies that we examined.

## Results

We performed immunoblots with plasma samples from 25 people with IBM, 25 people with other autoimmune muscle disease (10 dermatomyositis, 10 polymyositis, and 5 myasthenia gravis), and 15 healthy volunteers against normal human muscle lysates. While previous studies have probed myositis blood against proteins derived from non-muscle sources, such as those prepared from HeLa cells [10], we sought autoantibodies against proteins derived from human muscle.

**Figure 1:**
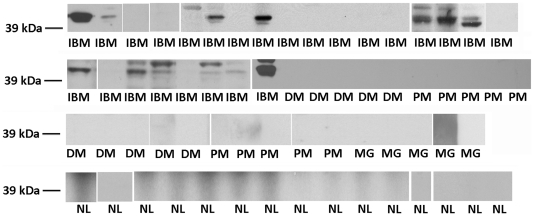
Circulating autoantibodies against a 43 kDa muscle autoantigen in inclusion body myositis (IBM). Images from all immunoblots are shown. Reactivity is present in 13 of 25 different inclusion body myositis (IBM) plasma samples, but in none of 10 dermatomyositis (DM), 10 polymyositis (PM), 5 myasthenia gravis (MG) or 15 normal volunteer (NL) plasma samples.

Immunoblots against normal human muscle lysates of blood samples from 65 people showed reactivity to an approximately 43 kDa muscle protein in 52% (13 of 25) of IBM samples and in no other autoimmune myopathy or healthy volunteer samples (0 of 40; p<0.0001 Fisher's exact test) (Figure 1). The detection of a 43 kDa muscle autoantigen thus had a sensitivity of 52% and specificity of 100% for IBM among 50 patients with autoimmune myopathies. The approximately 43-kDa band was sometimes (8 of 13 of positive samples) associated with a fainter nearby band, that may be seen with post-translational modification or partial protein degradation.

IBM is a disease of middle to late age; our patients with IBM were therefore older (mean age 68 years) than patients with other autoimmune myopathies (mean age 48 years), but the presence of the anti −43 kDa autoantibody did not appear age-associated. The mean age of the 13 IBM patients with anti-43-kDa autoantibodies (67 years) did not differ from the mean age of the 13 oldest control patients, none of whom had anti-43-kDa autoantibodies (64 years; p = 0.21 Mann-Whitney test). Even within the group of IBM, age played no role in the development of anti-43-kDA autoreactivity, as the mean age of IBM patients with autoreactivity (67 years) did not differ from those without autoreactivity (69 years; p = 0.76 Mann-Whitney test). Disease duration was not different between IBM patients demonstrating 43 kDa autoreactivity (6 years) and those without reactivity (8 years; p = 0.6 Mann-Whitney test). Gender (p = 0.21) and race (p = 1.0) were also not associated with the presence of the 43-kDa autoantibody when comparing all patients studied (Fisher's exact test for both analyses).

Treatment status did not appear to affect autoantibody detection, even though most patients with IBM were untreated while most patients with DM and PM received immunomodulating therapy. Within the group of IBM, the proportion of antibody positive untreated patients (12 of 21) did not differ from that of antibody positive treated patients (1 of 4; p = 0.32 Fisher's exact test). Analysis of data of all untreated patients showed statistically significant development of autoantibodies in IBM (12 of 21) but not in other disease controls (0 of 7; p = 0.01 Fisher's exact test for both comparisons) despite lack of treatment. For the 16 IBM patients tested for anti-nuclear antibodies (ANA) there was no relationship between 43 kDa autoreactivity and a positive test for ANA (p = 1 Fisher's exact test).

## Discussion

Autoimmune muscle injury in IBM has been widely believed to be mediated by cytotoxic T cell mechanisms alone (reviewed in [11]). The demonstration of antigen-stimulated plasma cell antibody production [8] and now a circulating IBM autoantibody against a 43 kDa muscle protein provides compelling evidence for humoral autoimmunity in IBM. Because of the principal of linked recognition (B cell aided maturation of T cell requires that both B cell immunoglobulin and T cell receptor recognize the same molecular complex), antigens to which autoantibodies are directed may also be candidates for T cell directed autoimmunity. The modest sensitivity (52%) but very high specificity (100%) of this autoantibody, in our group of IBM and other inflammatory myopathy patients, is a feature shared by many other autoantibodies in myositis [1] [12].

While previous studies have reported an association between IBM and the presence of various autoantibodies, thus suggesting an autoimmune process in IBM, none have been identified as disease specific [13] and their prevalence is less than that seen with DM and PM [3] [14] [15] [16] [17]. In a review of 99 patients with sporadic IBM, 43 (44%) had elevated titers of one or more of nine different non disease-specific autoantibodies [13]. While 8 of 16 (50%) of our IBM patients tested for autoantibodies where positive for ANA, there was no relationship between this positivity and the presence of 43 kDa autoreactivity.

The identity of the 43 kDa muscle autoantigen in IBM reported here, as well as its specificity to muscle tissue, remains to be determined. Technical aspects of myositis antigen identification have typically resulted in a several year lag between recognition of an autoantigen by its weight on immunoblots and its subsequent definitive identification. For example, recognition of a 140 kDa autoantigen in dermatomyositis [10] preceded its definitive identification as IFIH1 by 4 years [18]. The eventual determination of this IBM autoantigen's identity may provide understanding of the pathogenesis of IBM and potentially aid in its diagnosis.

## Materials and Methods

### Ethics Statement

All samples originating from patients were derived after informed written consent was obtained and under protocols approved by the Partners Human Research Committee (PHRC), the Institutional Review Board (IRB) of Partners Research Management that is responsible for overseeing all human subject research conducted by Partners-affiliated investigators (such as Brigham and Women's Hospital).

### Patient Samples

Plasma samples from 25 patients with IBM (mean/range age 69/50–87 years), 25 patients with other autoimmune myopathies (mean/range age 48/24–91), and 15 healthy volunteers (mean/range age 52/34–62 years) were probed against normal human muscle lysates. Diagnostic criteria for IBM, dermatomyositis, and polymyositis were as previously described [19]. In particular, all patients with IBM fulfilled European Neuromuscular Centre (ENMC) criteria for probable or definite IBM [20]. All healthy volunteers were required to have been free of any infectious or inflammatory diseases, the use of immunomodulatory agents or vaccination at least for six months prior to collection of blood sample. Detailed description about patients and normal control characteristics, including age, gender, racial background, disease and diagnostic workup characteristics, treatment status, treatment response and 43 kDa muscle protein autoreactivity can be found in the supplemental tables (Table S1 and Table S2, respectively).

### Preparation of gels and immunoblots

Muscle lysates were prepared and probed as previously described [19]. Briefly, 5 mg of cryostat sectioned normal human muscle was dounce homogenized in 200 µl of lysis buffer (containing 10 mM HEPES, 10 mM KCl, 1 mM EDTA, 0.1 mM EGTA, 10 mM DTT, 5 mM MgCl_2_ and Roche Complete Protease Inhibitor) and centrifuged at 2,000 g for 10 minutes at 4°C. The supernatant was removed and 30 µg loaded in individual wells of 4–12% NuPAGE Novex Bis-Tris Gels (cat# NP0322BOX, Invitrogen). Proteins were separated using SDS-PAGE and transferred to a nitrocellulose membrane. We used human plasma (1∶1000 dilution) as primary antibody and goat anti human-IgG HRP (cat# 31410, Pierce Biotechnology, 1∶60,0000 dilution) as secondary antibody, both for 1 hour at room temperature. Blots were developed using the SuperSignal West Pico kit (cat# 34077, Pierce Biotechnology, 5 min, room temperature).

### Statistical Analysis

To demonstrate the presence of selective immunoreactivity against a 43 kDa muscle protein in plasma of patients with IBM, compared with disease controls and healthy volunteers, as well as determining the effects of gender, treatment, race and ANA positivity on such reactivity we used the Fisher's exact test. We used the non-parametric Mann-Whitney test to determine the effect of age and disease duration on plasma immunoreactivity against the 43 kDa muscle protein.

## Supporting Information

Table S1 Patient characteristics for 25 inclusion body myositis (IBM), 10 dermatomyositis (DM), 10 polymyositis (PM) and 5 myasthenia gravis (MG) patients.
(XLS)

Table S2 Characteristics for 15 normal volunteers.
(XLS)
